# The brown fat-specific overexpression of RBP4 improves thermoregulation and systemic metabolism by activating the canonical adrenergic signaling pathway

**DOI:** 10.1038/s12276-025-01411-6

**Published:** 2025-03-03

**Authors:** Jong Yoen Park, Eun Sun Ha, Jimin Lee, Pierre-Jacques Brun, Yeri Kim, Sung Soo Chung, Daehee Hwang, Seung-Ah Lee, Kyong Soo Park

**Affiliations:** 1 Department of Molecular Medicine and Biopharmaceutical Sciences, Graduate School of Convergence Science and Technology, Seoul National University, Seoul, Republic of Korea; 2 School of Biological Sciences, Seoul National University, Seoul, Republic of Korea; 3 Department of Medicine, Institute of Human Nutrition, College of Physicians and Surgeons, Columbia University, New York, NY USA; 4 Biomedical Research Institute, Seoul National University Hospital, Seoul, Republic of Korea; 5 Bioinformatics Institute, Bio-MAX, Seoul National University, Seoul, Republic of Korea; 6 Genomic Medicine Institute, Medical Research Center, Seoul National University, Seoul, Republic of Korea; 7 ProGen Co. Ltd., 07789 Seoul, Republic of Korea

**Keywords:** Obesity, Genetic models

## Abstract

Retinol-binding protein 4 (RBP4), the sole specific carrier for retinol (vitamin A) in circulation, is highly expressed in liver and adipose tissues. Previous studies have demonstrated that RBP4 plays a role in cold-mediated adipose tissue browning and thermogenesis. However, the role of RBP4 in brown adipose tissue and its metabolic significance remain unclear. Here we generated and studied transgenic mice that express human RBP4 (hRBP4), specifically in brown adipocytes (UCP1-RBP4 mice), to better understand these uncertainties. When fed a chow diet, these mice presented significantly lower body weights and fat mass than their littermate controls. The UCP1-RBP4 mice also showed significant improvements in glucose clearance, enhanced energy expenditure and increased thermogenesis in response to a cold challenge. This was associated with increased lipolysis and fatty acid oxidation in brown adipose tissue, which was attributed to the activation of canonical adrenergic signaling pathways. In addition, high-performance liquid chromatography analysis revealed that plasma RBP4 and retinol levels were elevated in the UCP1-RBP4 mice, whereas their hepatic retinol levels decreased in parallel with a chow diet. Steady-state brown fat levels of total retinol were significantly elevated in the UCP1-RBP4 mice, suggesting that their retinol uptake was increased in RBP4-expressing brown adipocytes when fed a chow diet. These findings reveal a critical role for RBP4 in canonical adrenergic signaling that promotes lipid mobilization and oxidation in brown adipocytes, where the harnessed energy is dissipated as heat by adaptive thermogenesis.

## Transgenic mice reveal the role of RBP4 in fat metabolism

Brown adipose tissue, or brown fat, helps generate heat in our bodies. This study explores how retinol-binding protein 4 affects the ability of brown fat to burn energy. The researchers used mice genetically modified to produce more RBP4 in their brown fat. They found that these mice had lower body weight and better glucose control compared with normal mice. The study involved analyzing the mice’s fat tissues and measuring their energy use and fat breakdown. The researchers discovered that increased RBP4 in brown fat led to higher energy expenditure and improved fat metabolism. This suggests that RBP4 plays a role in activating brown fat and could help in managing obesity and related metabolic disorders. The findings indicate that targeting RBP4 in brown fat might be a potential strategy for treating obesity.

This summary was initially drafted using artificial intelligence, then revised and fact-checked by the author.

## Introduction

Brown adipose tissue (BAT), commonly referred to as brown fat, is a specialized type of adipose tissue found in mammals, including humans. Unlike white adipose tissue (WAT), which stores energy in the form of triglycerides (TGs), brown fat is primarily responsible for heat generation through a process called thermogenesis^1,2^. The unique function of brown fat is attributed to its high concentration of mitochondria and the presence of uncoupling protein 1 (UCP1) in the inner mitochondrial membrane. UCP1, a key biomarker of BAT activity, acts as a proton transporter that uncouples mitochondrial respiration from ATP synthesis and dissipates the proton gradient in the form of heat^3,4^.

The major signaling pathway for brown fat-specific thermogenic activity is β3-adrenergic signaling. Upon exposure to certain metabolic stimuli, such as cold, the norepinephrine released by the sympathetic nervous system mainly binds the β3-adrenergic receptor (β3-AR), which leads to an increase in the level of intracellular cAMP for the activation of protein kinase A (PKA)^5^. A variety of downstream targets of PKA, such as p38, cAMP response element-binding protein (CREB), and hormone-sensitive lipase (HSL), enhance most of the thermogenic responses of brown adipocytes, resulting in increased lipolysis, where stored TGs are broken down into free fatty acids (FFAs) and glycerol^6^. Lipolysis is a crucial step in the thermogenic processing of brown fat. The released FFAs not only act as obligatory activators of UCP1, promoting heat production, but also serve as metabolic substrates for fatty acid oxidation (FAO) within mitochondria^5^. FAO, also known as β-oxidation, involves the breakdown of FFAs to generate acetyl-CoA, which enters the citric acid cycle to produce reducing equivalents that drive ATP synthesis and contribute to the maintenance of the proton gradient necessary for thermogenesis^7^.

BAT-mediated thermogenesis protects against hypothermia and helps counteract obesity by expending energy. Previous studies have demonstrated the emergence of brown-like fat cells in WAT, called beige or brite adipocytes, under certain stimulatory conditions through a process known as browning^8,9^. Metabolically active BAT or beige adipocytes in adult humans are inversely correlated with body mass index, adiposity and insulin resistance. Thus, targeting BAT for interventions and understanding the molecular mechanisms underlying WAT browning have become attractive strategies for treating obesity^10^.

Retinol-binding protein 4 (RBP4), which is synthesized mainly in the liver, is the sole specific transporter of vitamin A in circulation^11^. Although its main function is related to vitamin A transport, RBP4 has been implicated in various metabolic processes and is expressed in both WAT and BAT, albeit to differing extents and potentially distinct roles^11^. As RBP4 has been proposed to function as an adipokine^12^, large observational studies involving diverse patient cohorts and animal studies have provided evidence for associations between elevated circulating and tissue RBP4 levels and the development of type 2 diabetes^12,13^, cardiovascular disease^14^ and non-alcoholic fatty liver disease^15,16^. Elevated expression and secretion of RBP4 in white adipocytes of mice led to adverse metabolic consequences, including insulin resistance and hepatic steatosis^17^. In contrast, the role of RBP4 in BAT remains poorly understood. Previous studies have reported an association between vitamin A status and obesity in animal models^18–21^ and human subjects^20,22,23^. Compared with control, mice fed a vitamin A-deficient diet developed increased adiposity^18^, and rats supplemented with vitamin A presented reduced adiposity and improved metabolic parameters^21^. Similarly, individuals with lower serum vitamin A levels had a greater prevalence of obesity than those with higher vitamin A levels; conversely, individuals with higher dietary vitamin A intake had a lower risk of developing obesity over time than those with lower vitamin A intake, highlighting the importance of maintaining optimal vitamin A levels for obesity prevention^22,23^. Thus, vitamin A is known for its benefits in preventing metabolic diseases. Recently, Kiefer and colleagues demonstrated that cold stimulation in mice and humans increases the levels of circulating retinol and its plasma transporter, RBP4. They demonstrated in *Rbp4*-knockout mice that intact retinoid transport is essential for cold-induced adipose tissue browning and adaptive thermogenesis^24^. Studies on retinol-induced WAT browning have established that retinol promotes thermogenic gene expression and mitochondrial respiration in vitro and in vivo^25,26^.

Both types of adipose tissue store vitamin A derivatives (collectively known as retinoids) and play a role in retinoid metabolism^27^. Most actions of vitamin A are understood to be mediated by the active metabolite all-*trans* retinoic acid (ATRA), which acts through retinoic acid receptors and retinoid X receptors (RXRs). ATRA functions as an anti-adipogenic agent in adipocytes and is a powerful inducer of UCP1 expression, which is responsible for thermogenic function^28^. Given that cold exposure regulates systemic RBP4 and retinol levels^24^, RBP4 in brown fat facilitates retinol uptake from the circulation and promotes BAT activation and thermogenic programs in WAT. In the present study, we aimed to explore the possible effect of RBP4 on brown fat function by assessing a transgenic mouse model that expresses RBP4 specifically in brown adipocytes to further decipher the molecular mechanisms underlying RBP4 activity.

## Materials and methods

### Animals

The mice carrying the pROSA26-1-STOP-hRBP4-eGFP transgene (human RBP4 (hRBP4) knock-in mice) used in our studies were kindly provided by Dr. William S. Blaner (Columbia University). The characteristics of these mice have been previously described in detail^17^. To generate brown fat-specific hRBP4 (UCP1-RBP4) transgenic mice, hRBP4 knock-in mice were bred with UCP1-Cre mice. Littermates lacking the UCP1-Cre transgene served as the controls. After weaning, the mice were maintained on a standard chow diet (38057, Purina Animal Nutrition LLC) that provided 28 IU/g diet vitamin A. The mice were maintained on a 12 h light/dark cycle and had ad libitum access to food and water. All experiments were approved by the Seoul National University Bundang Hospital Institutional Animal Care and Use Committee in accordance with the Guide for Experimental Animal Research of the Laboratory Animals (permit number 62-2021-070). The results of the animal experiments are provided in the Supplementary Information.

### HPLC analysis of retinol and retinyl esters

Total plasma and tissue retinol (retinol and retinyl esters) levels were analyzed via high-performance liquid chromatography (HPLC) according to our previously described standard protocol^29^. Briefly, after protein denaturation in 100% ethanol equal to the volume of a known internal standard, the total retinol was extracted from the plasma or tissue homogenates into hexane. After hexane evaporation, the lipid residues, including retinol and retinyl esters, were dissolved in benzene and separated on a Symmetry C18 column (Waters). To quantify the extracted retinoids, the areas under the absorbance peaks at 325 nm were measured and adjusted to recover an internal standard (retinyl acetate, Sigma-Aldrich).

### FAO

BATs were homogenized in ice-cold mitochondrial isolation buffer (250 mmol/l sucrose, 10 mmol/l Tris–HCl and 1 mmol/l EDTA). After 2 h of incubation with 0.2 mmol/l [^14^C] palmitate, ^14^CO_2_- and ^14^C-labeled acid-soluble metabolites were quantified via a liquid scintillation counter. The radioactivity of each lysate was normalized through protein quantification.

### In vivo, ex vivo and in vitro lipolysis assays

For the in vivo lipolysis assay, blood was collected at 0, 2 and 4 h after the β3-AR agonist, CL316,243 (1 mg/kg body weight; Sigma-Aldrich) was injected intraperitoneally into the overnight-fasted mice. During this period, the mice were housed at room temperature without food; however, water was readily available. For the ex vivo and in vitro lipolysis assays, 20 mg of BAT, 30 mg of inguinal or epididymal WAT explants, or the differentiated brown adipocytes were cultured in Krebs-Ringer HEPES buffer (115 mM NaCl, 5.9 mM KCl, 1.2 mM MgCl_2_, 1.2 mM NaH_2_PO_4_, 2.5 mM CaCl_2_, 25 mM NaHCO_3_ and 12 mM HEPES, pH 7.4) with 2% fatty acid-free bovine serum albumin (Sigma-Aldrich) and 5 mM glucose. The medium was collected to determine the FFA and glycerol levels, which were normalized to the protein concentrations.

### RNA sequencing and analysis

Total RNA was isolated from the BAT of the UCP1-RBP4 and matched control mice fed a chow diet (*n* = 3) for paired-end DNA sequencing. The cDNA library was constructed via the TruSeq Stranded mRNA LT Sample Prep kit after ribosomal RNA was eliminated and then sequenced via the Illumina NovaSeq 6000 platform (Macrogen). Adapter sequences were trimmed, reads of low quality or short lengths were removed via Trimmomatic (version 0.38)^30^ and the resulting reads were aligned to the mm10 reference genome via HISAT2 (version 2.1.0)^31^. The mapped reads were used to quantify gene expression levels via StringTie (version 2.1.3b)^32^.

### Identification of DEGs

Expressed genes were initially identified as those with fragments per kilobase of transcript per million mapped reads (FPKM) values greater than 1 in at least one sample. The FPKM values of these genes were subsequently transformed to log_2_(FPKM + 1) values and normalized via the quantile normalization method^33^. Using the normalized expression levels, we identified the differentially expressed genes (DEGs) between the UCP1-RBP4 and control mice via a previously established empirical *t*-test method^34^. Briefly, for each gene, *t*-statistic values were calculated by comparing the UCP1-RBP4 and control mice. An empirical null distribution of the *t*-statistic value was estimated by performing 1,000 random permutations of all the samples, and the multiple testing-corrected *P* values of *t*-statistic values were computed via the estimated empirical distribution (two-sided test). Finally, the DEGs were defined as those with an adjusted *P* value of <0.05 and an absolute log_2_ (fold change) >0.336 (5% percentile).

### Enrichment analysis of cellular pathways

To identify the pathways represented by the DEGs, enrichment analysis of cellular pathways was performed via ConsensusPathDB^35^. Specifically, pathways related to phenotypes associated with RBP4 overexpression in BAT were selected based on a significance threshold of *P* < 0.05 obtained from the expression analysis systematic explorer test.

The Supplementary Information provides detailed descriptions of other experimental protocols employed in this study, including metabolic measurements, biochemical analyses, immunohistochemistry, cell culture, western blotting, quantitative real-time PCR, seahorse assays and statistical analyses.

## Results

### Endogenous mRNA expression of RBP4 in brown fat is increased after thermogenic stimulation

**Fig. 1:**
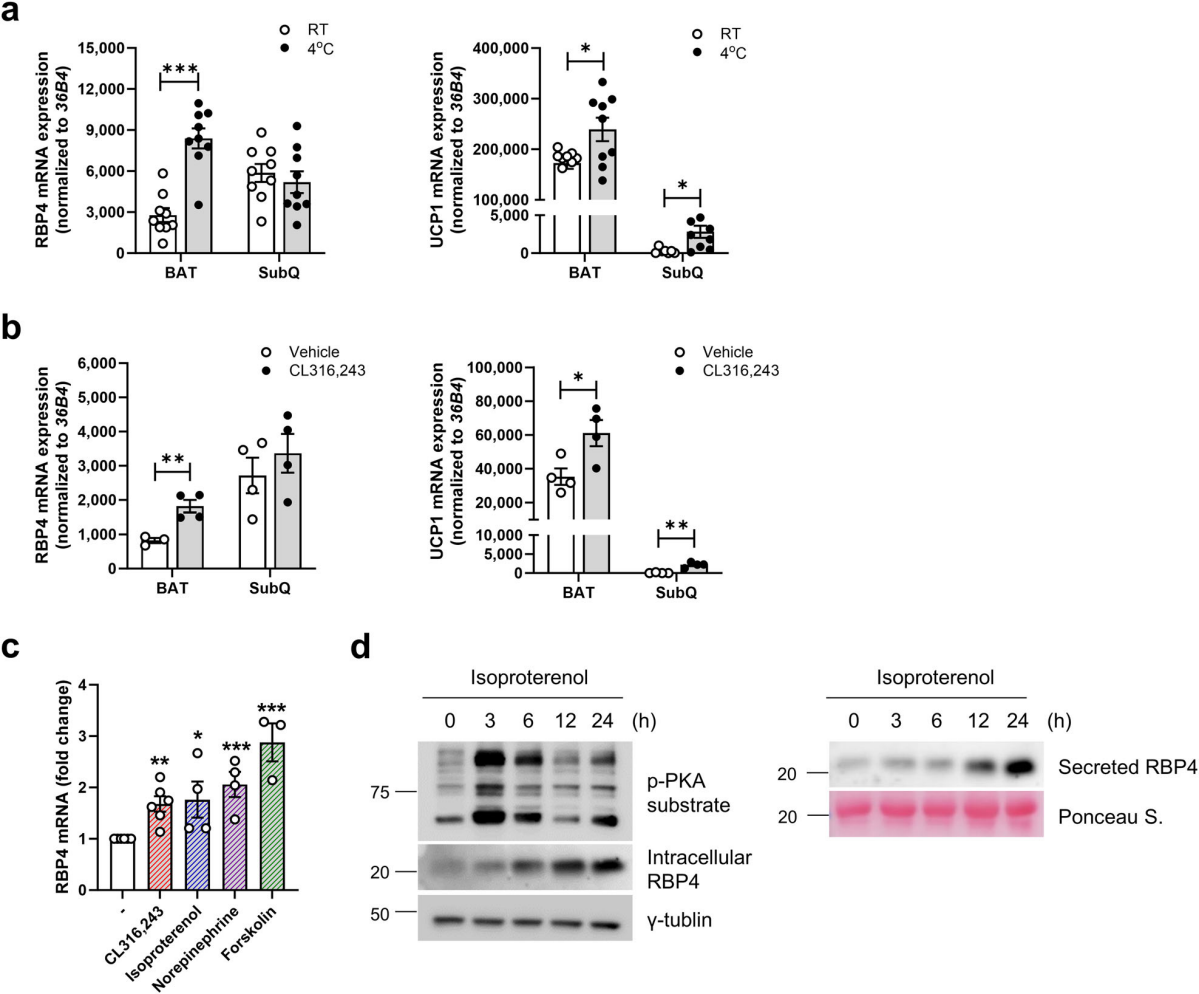
Endogenous RBP4 mRNA expression in brown fat increases after thermogenic stimulation. **a**, **b** C57BL/6 mice were exposed to 4 °C ambient temperature for 3 h (*n* = 9 mice per group) (**a**) or were intraperitoneally injected with 1 mg/kg body weight CL316,243 for 24 h (*n* = 3–4 mice per group) (**b**). Endogenous *Rbp4* and *Ucp1* mRNA levels in BAT and SubQ were determined via qPCR. **c** RBP4 mRNA expression was determined in immortalized brown adipocytes differentiated and exposed to 0.1 μM CL316,243, 0.1 μM isoproterenol, 1 μM norepinephrine or 10 μM forskolin for 6 h (*n* = 3–6 independent samples). **d** Cells were treated with 0.1 μM isoproterenol at the indicated time points, and the cell lysates and culture media were subjected to western blotting for phospho-PKA substrate and RBP4. The data are presented as the means ± s.e.m. *P < 0.05, **P < 0.01 and ***P < 0.001.

We observed basal RBP4 mRNA and protein levels in three different adipose tissues. RBP4 mRNA and protein were highly expressed in visceral WAT (VISC), followed by subcutaneous WAT (SubQ) and BAT (Supplementary Fig. 1a,b). One published study reported that *Rbp4* gene expression is induced in brown fat from mice exposed to cold^36^. However, another study did not support this finding; it reported that cold stimulation in mice and humans increased circulating retinol and RBP4 concentrations but not its concentration in brown fat^24^. Thus, we investigated the effects of cold-mediated RBP4 induction on BAT expression in mice. Cold exposure (6 h at 4 °C) and stimulation with a selective β3-AR agonist (CL316,243) caused a notable increase in *Rbp4* mRNA levels in BAT, whereas no effects were observed in SubQ adipose tissue (Fig. 1a, b). As expected, cold exposure and CL316,243 stimulation dramatically increased *Ucp1* mRNA expression in both BAT and SubQ fat. In addition, other thermogenic stimuli, such as isoproterenol, norepinephrine and forskolin, as well as CL316,243, significantly increased *Rbp4* mRNA expression in immortalized brown adipocytes, thus confirming the sensitivity of *Rbp4* to thermogenic stimuli in BAT (Fig. 1c). In line with the elevation in *Rbp4* mRNA levels, RBP4 protein levels were increased in both brown adipocytes and the culture medium following isoproterenol-induced PKA activation (Fig. 1d). This observation indicates that thermogenic stimulation leads to a significant time-dependent increase in RBP4 protein abundance.

### Brown adipocyte-specific RBP4 overexpression improves glucose homeostasis

**Fig. 2:**
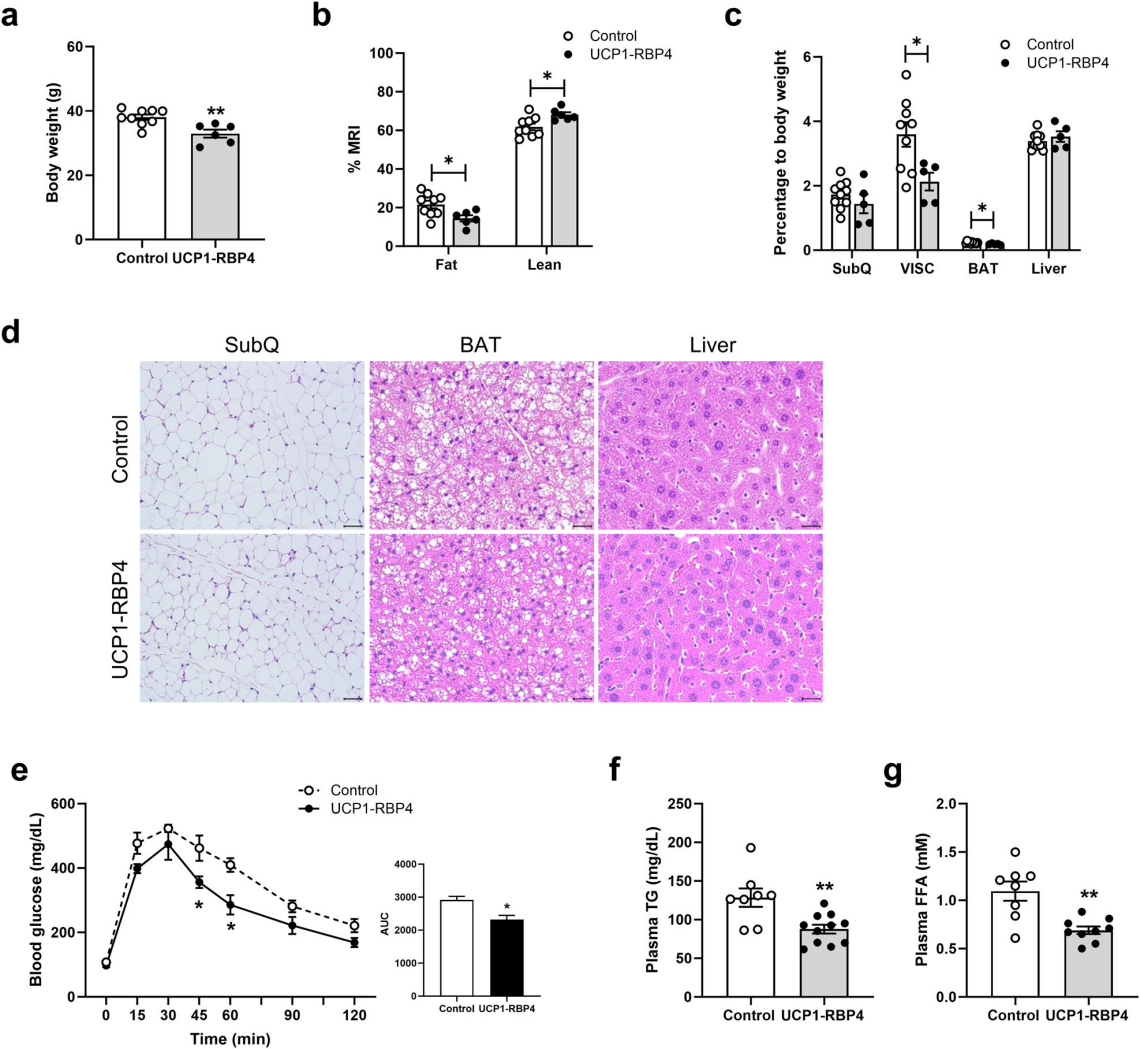
UCP1-RBP4 mice gain less body weight and show better glucose tolerance than matched littermate controls. **a** UCP1-RBP4 mice (6–7-month-old males) fed a chow diet had significantly lower body weights than matched controls. **b** Magnetic resonance imaging was performed on 6-month-old mice. **c** The weights of SubQ, VISC, BAT and liver tissues normalized to body weight for 6–7-month-old control and UCP1-RBP4 mice fed a chow diet. **d** Hematoxylin and eosin staining of the SubQ, BAT and liver revealed that the sizes of the lipid droplets in the SubQ, BAT and liver were significantly smaller in UCP1-RBP4-treated mice than in control mice. Scale bar, 50 μm; original magnification, 200× for SubQ and 400× for BAT and liver. **e** Time course showing blood glucose levels in response to an intraperitoneal challenge of glucose (2 g/kg, body weight) for matched 5-month-old male UCP1-RBP4 and control mice fed a chow diet throughout life. The area under the glucose clearance curve (AUC) (right) for UCP1-RBP4 mice was lower than that for matched control mice. **f**, **g** Fasting plasma TG (**f**) and nonesterified fatty acid (FFA) (**g**) levels following overnight fasting were also significantly lower in 6–7-month-old male UCP1-RBP4 mice than the control mice. The data are presented as the means ± s.e.m. from 6–10 mice in each group. *P < 0.05 and **P < 0.01.

To study the functional effects of RBP4 in brown fat, we generated transgenic mice that could specifically express hRBP4 in brown adipocytes (UCP1-RBP4). To verify transgene expression, various tissue extracts were analyzed for hRBP4 expression via western blotting. The *hRBP4* transgene was selectively expressed in BAT (Supplementary Fig. 1c). The UCP1-RBP4 mice presented increased total RBP4 (mRBP4 + hRBP4) mRNA and protein levels in BAT compared with the levels of endogenous mRBP4, as determined through qPCR and western blotting analyses, respectively (Supplementary Fig. 1d,e). The 3–4-month-old male UCP1-RBP4 mice had lower body weights than the control mice when fed a chow diet; however, this trend was not statistically significant (data not shown). However, the 6–7-month-old male UCP1-RBP4 mice fed a chow diet had significantly lower body weights than their matched controls (Fig. 2a). Magnetic resonance imaging analysis revealed that decreased body weight was associated with decreased body fat and increased lean mass (Fig. 2b). A significant decrease in fat mass was observed in the VISC and BAT but not in the SubQ or liver (Fig. 2c). Hematoxylin and eosin-stained sections of the SubQ, BAT and liver revealed that the size of the lipid droplets in these tissues was notably smaller in the UCP1-RBP4 mice than in the control mice (Fig. 2d). Fasting hepatic TG levels were slightly but significantly decreased in the 6–7-month-old male UCP1-RBP4 mice, whereas brown fat TG levels were not affected (Supplementary Fig. 1f,g). We observed improved glucose tolerance in the 5-month-old male UCP1-RBP4 mice fed a chow diet (Fig. 2e). Notably, the UCP1-RBP4 mice presented significantly lower areas under the glucose clearance curves following an intraperitoneal glucose challenge. Fasting plasma TG and nonesterified FFA levels also differed between the matched UCP1-RBP4 and control mice (Fig. 2f, g). Consistent with the body weights, fasting plasma leptin levels were significantly lower in the chow-fed UCP1-RBP4 mice than in the control mice. However, the fasting plasma adiponectin levels were comparable between the groups (Supplementary Fig. 1h,i).

### Brown adipose RBP4 overexpression affects retinoid homeostasis in chow-fed mice

**Fig. 3:**
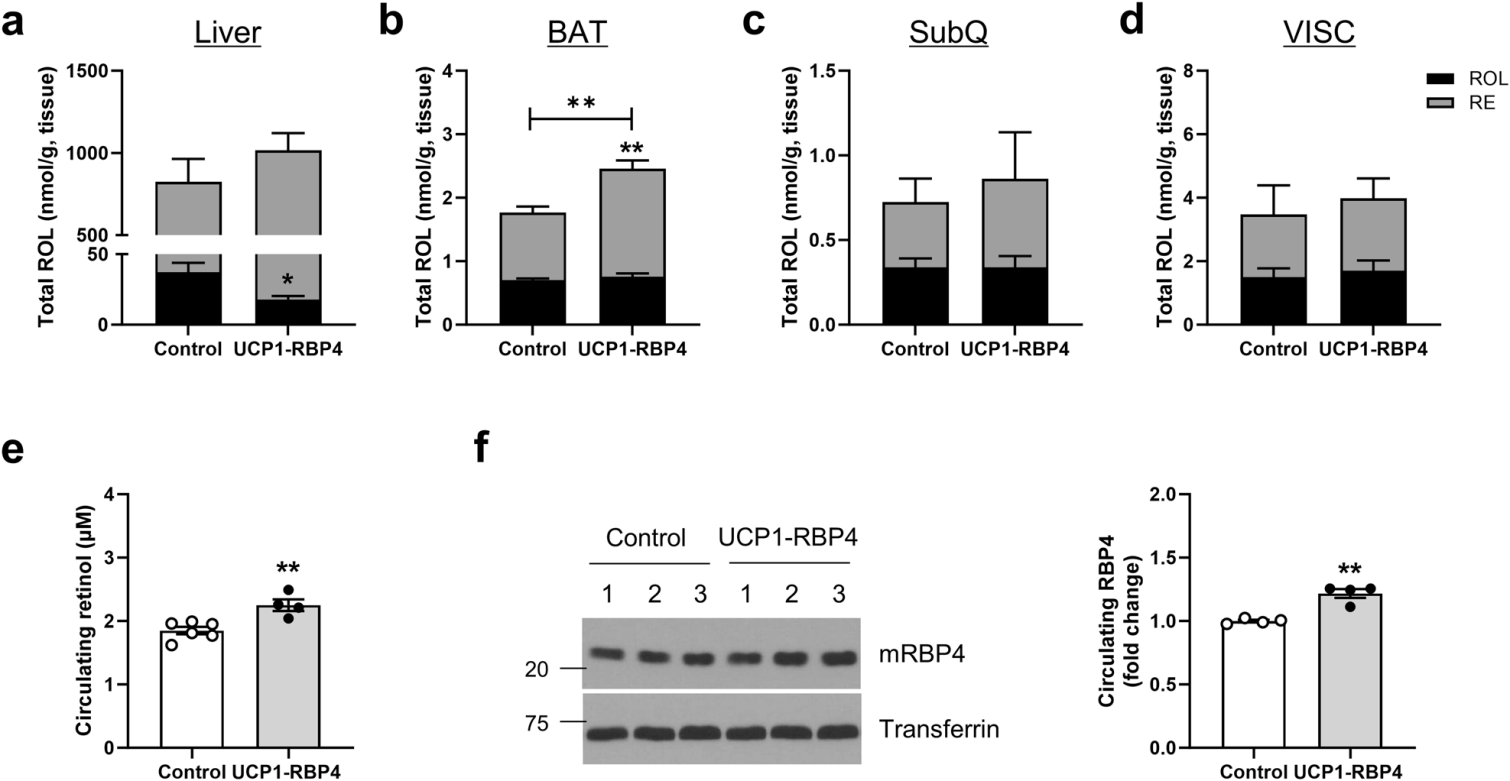
The overexpression of brown adipose RBP4 affects retinoid homeostasis in chow-fed mice. Tissues were collected from 6-month-old male UCP1-RBP4 mice and matched control mice fed a chow diet. **a**–**e** Total retinol levels (ROL, black bar) + retinyl ester (RE, gray bar) in the liver (**a**) BAT (**b**) SubQ (**c**) VISC (**d**) and plasma (**e**) were quantified via HPLC. **f** Plasma RBP4 levels were analyzed by western blotting. RBP4-specific bands were quantified via ImageJ (*n* = 4). The data are presented as the mean ± s.e.m. from 6–10 mice in each group. *P < 0.05 and **P < 0.01.

As RBP4 is the sole specific transport protein for retinol in circulation, we evaluated parameters associated with the maintenance of retinoid homeostasis in age-matched male UCP1-RBP4 and control mice fed a standard chow diet containing 28 IU/g vitamin A. HPLC demonstrated that hepatic retinol levels in the UCP1-RBP4 mice were significantly lower than those in the control mice fed a chow diet by approximately 50% (Fig. 3a). This increase was accompanied by a significant increase in the plasma retinol and RBP4 levels in the chow-fed UCP1-RBP4 mice (Fig. 3e, f). In accordance with the increased plasma RBP4 levels, brown adipose RBP4 overexpression induced *Rbp4* mRNA expression in the livers of the mice. This finding is consistent with other findings in which cold exposure-mediated *Rbp4* mRNA increases were observed in the livers of mice^24^ (Supplementary Fig. 2a). Other hepatic genes associated with the retinoid machinery, such as *Lrat*, *Rbp1*, *Rdh1*, *Rdh10*, *Raldh1*, *Cyp26a1* and *Cyp26b1*, were not altered by brown adipose RBP4 overexpression (Supplementary Fig. 2b). Genes involved in lipid metabolism, such as *Srebp1c*, *Dgat1*, *Dgat2*, *Atgl*, *Hsl*, *Lpl* and *Cd36*, were also unaffected in the liver following the overexpression of brown adipose RBP4 (Supplementary Fig. 2c). Interestingly, retinyl ester levels in the BAT of the UCP1-RBP4 mice fed a chow diet significantly increased, affecting total retinol (all-*trans*-retinol and retinyl esters) levels in BAT (Fig. 3b). However, the total retinol levels did not differ between the SubQ and VISC of the two genotypes (Fig. 3c, d). These findings indicate that a modest elevation in brown adipocyte RBP4 expression regulates systemic RBP4 and retinol levels and affects retinoid redistribution between the liver and BAT.

### Brown adipocyte-specific RBP4 overexpression increases energy expenditure and FAO in BAT, inducing WAT browning

**Fig. 4:**
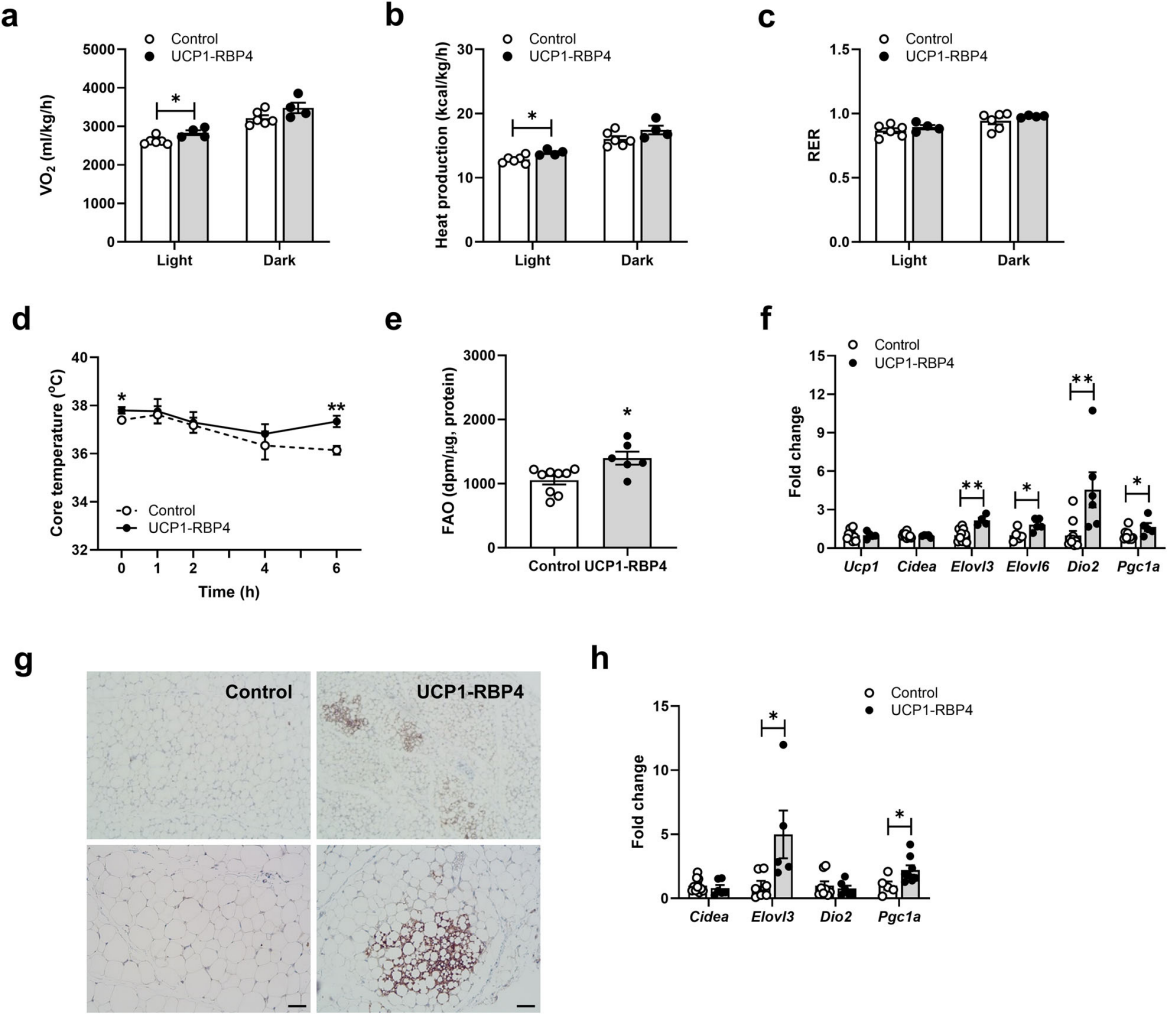
UCP1-RBP4 mice have increased energy expenditure and FAO in BAT, resulting in WAT browning. **a**, **b** Calorimetry studies at room temperature (22 °C) of 6–7-month-old UCP1-RBP4 mice fed a chow diet throughout their lifetime established that the UCP1-RBP4 mice consumed significantly more O_2_ during the light cycle but not the dark cycle than control mice (**a**) and generated significantly more heat during the light cycle compared with control mice (**b**). **c** The RER of UCP1-RBP4 mice was similar to that of control mice. **d** The core body temperature in control and UCP1-RBP4 mice was measured at room temperature (22 °C) and during 6 h of exposure to cold (4 °C). **e** FAO measured in the BAT of mice. **f** mRNA expression of known BAT markers was determined by qPCR. **g** Immunohistochemical images of the SubQ-embedded UCP1 sections acquired with an ECLIPSE Ci-L microscope. Magnification, 100× (top) and 200× (bottom). Scale bar, 50 μm. **h** mRNA levels of several classical BAT markers were measured in SubQ of chow-fed control and UCP1-RBP4 mice via qPCR. The data are presented as the mean ± s.e.m. and were obtained from 5–7 mice in each group. *P < 0.05 and **P < 0.01.

Next, we investigated the mechanism of decreased body weight and adiposity and the functional effects of altered retinoid homeostasis in the overexpression of BAT-specific RBP4. Indirect calorimetry studies at room temperature were performed on 6–7-month-old UCP1-RBP4 mice fed a chow diet throughout their lifetime. The results showed that they consumed significantly more O_2_ during the light cycle but not the dark cycle (Fig. 4a) and generated significantly more heat during the light cycle than their matched controls (Fig. 4b). The energy expenditure rates of the UCP1-RBP4 mice displayed a modest yet significant increase compared with those of the control mice. The UCP1-RBP4 mice had a respiratory exchange ratio (RER) similar to the control mice (Fig. 4c). No significant differences in food intake or physical movements were observed (data not shown). As uncoupled respiration dissipates energy through heat production, we measured the core body temperature of the control and UCP1-RBP4 mice at room temperature (22 °C) and during 6 h of cold exposure (4 °C) to assess brown fat activity directly. Notably, at 22 °C (0 h), core body temperatures differed between the genotypes and at 4 °C, the UCP1-RBP4 mice were completely protected against cold exposure, indicating increased thermogenic activity (Fig. 4d). In addition, we observed increased FAO in the BAT of the UCP1-RBP4 mice (Fig. 4e). To characterize brown fat in the presence of RBP4, we measured the mRNA expression of known BAT markers, including *Ucp1*, *Cidea*, *Elovl3*, *Elovl6*, *Dio2* and *Pgc1a. Ucp1* mRNA levels remained unchanged in BAT, but the expression of other BAT marker genes, such as *Elovl3*, *Elovl6*, *Dio2* and *Pgc1a*, significantly increased in the BAT of the chow-fed UCP1-RBP4 mice (Fig. 4f). Next, we investigated whether activated BAT function might affect white fat browning and explain its systemic metabolic effect. According to the immunohistochemistry images, the lipid droplet size and adipocyte morphology appeared BAT-like, in part, in the SubQ of the UCP1-RBP4 mice, and the UCP1 protein content was locally and markedly increased compared with that in the control adipose tissue (Fig. 4g). The mRNA levels of several classical BAT markers were significantly increased in the SubQ of the chow-fed UCP1-RBP4 mice (Fig. 4h), suggesting that BAT-specific RBP4 overexpression may activate a thermogenic program in WAT.

### RBP4 positively regulates thermogenesis by activating the canonical adrenergic signaling pathway and enhances fat lipolysis in BAT

**Fig. 5:**
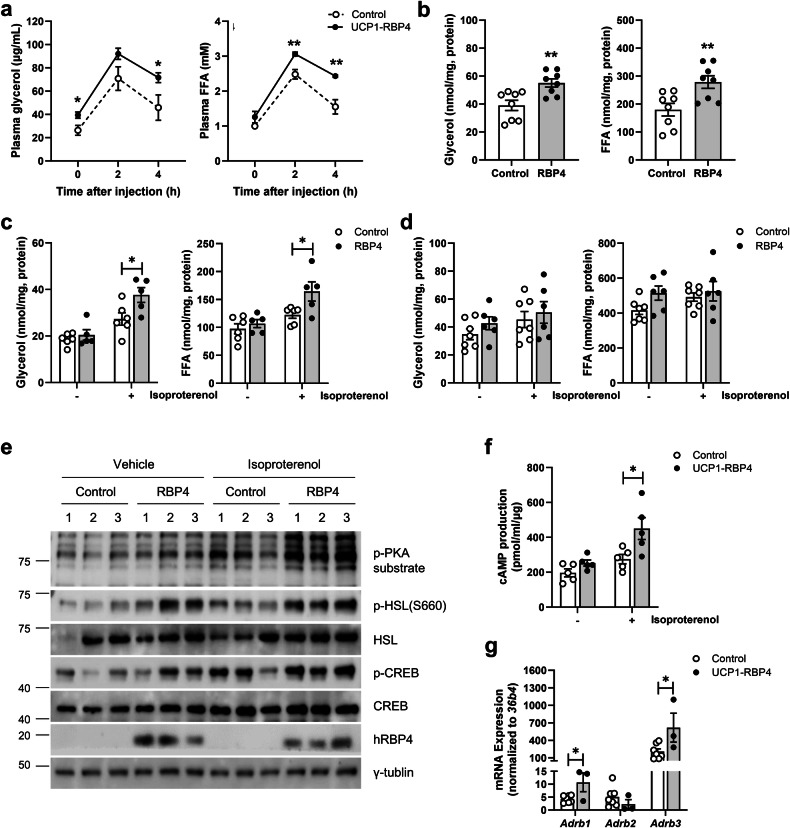
The overexpression of brown adipose RBP4 increases fat lipolysis and FAO. **a** Plasma glycerol and FFA levels were measured at the indicated time points in 3-month-old male mice following injection of CL316,243 at 1 mg/kg body weight. **b**–**e** Ex vivo lipolysis assay in BAT (**b**) SubQ (**c**) and VISC (**d**) isolated from 3-month-old male control and UCP1-RBP4 mice. **e** Western blots of lysates from primary brown adipocytes isolated from UCP1-RBP4 mice and matched controls, with or without 0.1 μM isoproterenol. **f**, **g** cAMP levels (**f**) and mRNA expression of *Adrb1*, *Adrb2* and *Adrb3* (**g**) measured in the BAT of control and UCP1-RBP4 mice at 5 months of age. The data are presented as the mean ± s.e.m. and were obtained from 5–7 mice in each group. *P < 0.05.

We investigated the underlying mechanism by which RBP4 regulates adipose thermogenic activity in BAT. CL316,243 drives lipolysis via the canonical adrenergic receptor–cAMP–PKA pathway. The fatty acids released during adrenergic stimulant-induced lipolysis act as triggers necessary for UCP1 activation and serve as metabolic substrates that fuel thermogenic respiration^5^. To evaluate systemic lipolysis in the UCP1-RBP4 mice, plasma glycerol and FFA levels were measured at 0, 2 and 4 h after the intraperitoneal injection of CL316,243. Plasma glycerol levels were significantly greater at basal levels and 4 h after CL316,243 injection in the 3-month-old male UCP1-RBP4 mice compared with the control mice. Plasma FFA levels in the transgenic mice were also greater at the 2 and 4 h time points compared with those of the control mice (Fig. 5a). Additionally, we observed significantly elevated rates of basal glycerol and FFA release in ex vivo brown fat explants from the 3-month-old male UCP1-RBP4 mice compared with the control mice (Fig. 5b). Importantly, the basal lipolysis rates of white fat explants from the SubQ and VISC WAT depots of the UCP1-RBP4 mice (Fig. 5c, d), where higher RBP4 expression was detected than that in BAT (Supplementary Fig. 1a,b), did not differ from those of the controls (Fig. 4c, d). However, when cold stimulation was induced by isoproterenol, the rate of lipolysis, as measured through glycerol and FFA release, significantly increased only in SubQ (Fig. 5c, d). Consistent with the expression of classical BAT markers in SubQ (Fig. 4g, h), this result suggests the potential for WAT browning and the presence of beige fat in the SubQ of UCP1-RBP4 mice in response to cold stimulation. Furthermore, RBP4-overexpressing brown adipocytes derived in vitro from the stromal vascular cell fraction presented increased levels of PKA activation when a pan-PKA substrate antibody that recognizes the RRXS/T motif under both basal and isoproterenol-stimulated conditions was used (Fig. 3e). We also observed elevated levels of HSL phosphorylation at S660 and CREB phosphorylation in RBP4-overexpressing brown adipocytes, before and after the cold stimulation (Fig. 5e). Next, we investigated whether the increased levels of PKA activation observed in RBP4-overexpressing brown fat were caused by increased cAMP levels. Compared with those in control cells, RBP4-overexpressing brown fat tended to have increased cAMP levels in the basal state, whereas after the cold stimulation, the cAMP levels in RBP4-overexpressing brown fat increased by ~100% (Fig. 5f). These findings strongly suggest that RBP4 potentiates cAMP production, thereby positively regulating PKA activation. This increased cAMP production was directly attributed to increased levels of components of adrenergic signaling pathways, as evidenced by changes in the mRNA levels of the adrenergic receptors *Adrb1* and *Adrb3* in RBP4-overexpressing brown adipocytes (Fig. 5g).

**Fig. 6:**
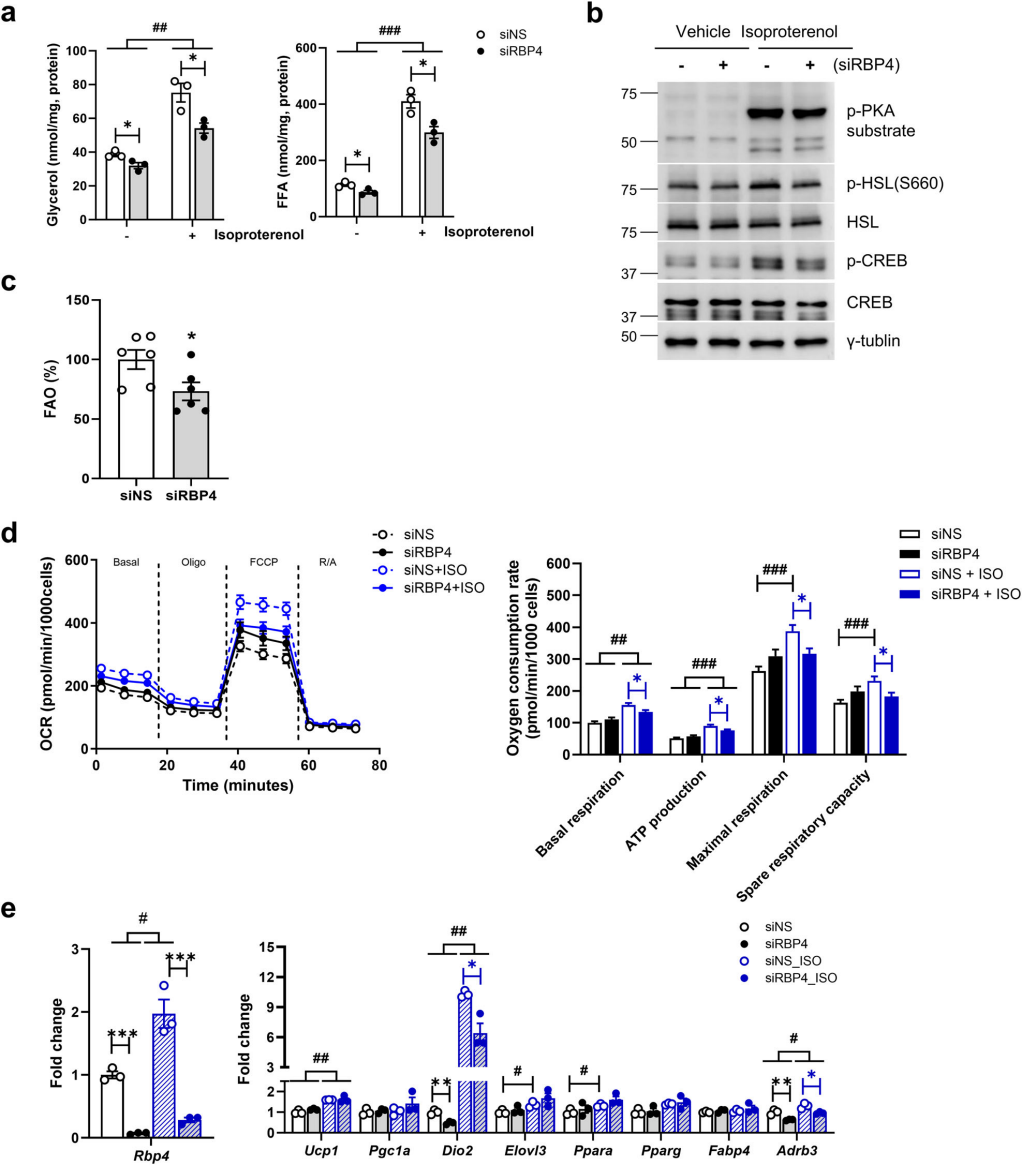
The downregulation of RBP4 confirms the alteration of RBP4-mediated lipolysis, FAO and mitochondrial function in brown adipocytes. **a** In vitro lipolysis was conducted in differentiated adipocytes with control or RBP4 repression, with or without isoproterenol (*n* = 3 independent samples). **b** Western blots were performed on cell lysates obtained from differentiated RBP4-repressed brown adipocytes, both with and without isoproterenol (*n* = 3 independent experiments). **c** FAO was measured in RBP4-repressed brown adipocytes (*n* = 6 independent samples). **d** The OCR was measured via a seahorse assay in the presence and absence of 0.1 μM isoproterenol for 24 h (left). OCR parameters were quantified for each group (right) (*n* = 5–6 independent samples). **e** The mRNA expression levels of genes associated with brown fat activation were measured via qPCR (*n* = 3). The data are presented as the mean ± s.e.m. *P < 0.05 and **P < 0.01 between genotypes. ^#^*P* < 0.05, ^##^*P* < 0.01 and ^###^*P* < 0.001 between vehicle- and isoproterenol-treated cells.

To assess whether increased fat mobilization in the UCP1-RBP4 mice was cell autonomous, we conducted in vitro lipolysis assays using *Rbp4*-knockdown brown adipocytes. Compared with the control cells, the *Rbp4*-knockdown brown adipocytes secreted less glycerol and FFAs in the basal and isoproterenol-induced states (Fig. 6a). Additionally, β3-adrenergic signaling, as evidenced by the phosphorylation of HSL, CREB and PKA substrates, was attenuated in the *Rbp4*-knockdown brown adipocytes (Fig. 6b). Moreover, compared with the control brown adipocytes, the *Rbp4*-knockdown brown adipocytes presented decreased FAO and oxygen consumption rates (OCRs) (Fig. 6c, d), accompanied by reduced mRNA expression of *Dio2* and *Adrb3* (Fig. 6e). These results strongly support the conclusion that RBP4 positively regulates BAT thermogenesis in vivo.

### Transcriptome analysis provides a network model for the regulation of lipid metabolism and thermogenesis by RBP4

**Fig. 7:**
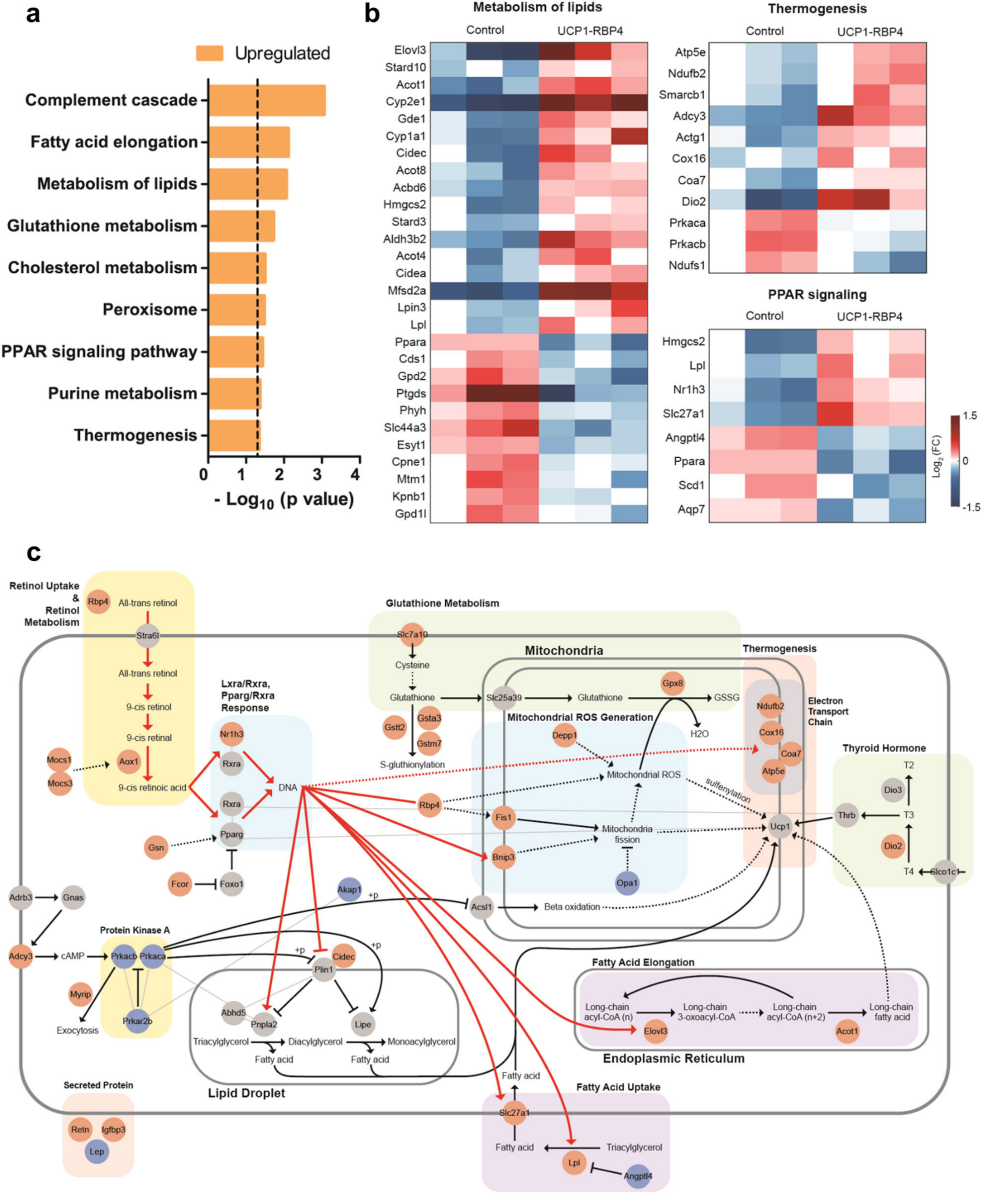
Transcriptome analysis provides a network model for RBP4-mediated regulation of lipid metabolism and thermogenesis. **a** A bar graph showing the cellular pathways significantly enriched with upregulated genes in UCP1-RBP4 mice compared with control mice. The enrichment significance is presented as the −log_10_(*P* value), where *P* is the enrichment *P* value from the expression analysis systematic explorer test. **b** Heat maps illustrating the upregulated and downregulated patterns of genes involved in thermogenesis, lipid metabolism and the PPAR signaling pathway. The color bar depicts the range of log_2_ fold changes in gene expression levels relative to their median levels. **c** A network model showing that RBP4 regulates genes involved in lipid metabolism and thermogenesis via NR1H3–RXRA transcriptional regulation. The solid lines represent direct activation or repression, whereas the dashed lines denote indirect effects. The color of the nodes indicates their upregulation (red), downregulation (blue) or no significant change (gray) in the UCP1-RBP4 mice compared with control mice.

To evaluate the metabolic role of RBP4 in BAT at the genome-wide level, we analyzed global transcriptional changes by performing bulk mRNA sequencing of the BAT of age- and sex-matched control and UCP1-RBP4 mice fed a standard chow diet. A total of 570 genes were identified as DEGs, including 281 upregulated genes and 289 downregulated genes, in UCP1-RBP4. Pathway enrichment analysis revealed the upregulation of thermogenesis and lipid metabolism pathways as well as the PPAR signaling pathway regulating lipid degradation in the UCP1-RBP4 mice (Fig. 7a), which is consistent with the findings shown in Fig. 5. The upregulated genes involved in these pathways are shown in Fig. 7b. To understand how these upregulated genes are collectively regulated by RBP4, we reconstructed a network model describing the interactions among RBP4 and these upregulated genes (Fig. 7c). Network analysis revealed that the overexpression of RBP4 upregulated *AOX1* and *MOCS1/3*, resulting in increased 9-*cis* retinoic acid production, subsequently activating Nr1h3–Rxra or Pparg–Rxra transcription factor complexes. Moreover, *NR1H3*, along with its regulators *GSN* and *FCOR*, was upregulated by RBP4 overexpression. The activation of these transcription factor complexes subsequently increased lipid metabolism and thermogenesis by upregulating (1) *ELOVL3* and *ACOT1*, which are involved in the biosynthesis of very long-chain fatty acids associated with cold-induced thermogenesis; (2) *SLC27A1* and *LPL*, which are involved in fatty acid uptake; (3) *CIDEC*, which is involved in lipolysis; and (4) *NDUFB2*, *COX16*, *COA7* and *ATP5E*, which are involved in oxidative phosphorylation and thermogenesis. Overall, the network model provides potential regulatory links between the overexpression of RBP4 and its observed effects on thermogenesis in BAT.

## Discussion

The present study demonstrated that brown adipose RBP4 acts as a positive regulator of thermogenesis based on our characterization of a BAT-specific RBP4-overexpressing mouse model. These findings elucidate the systemic metabolic effects observed in UCP1-RBP4 mice. We observed significant increases in circulating RBP4 and retinol levels accompanied by decreased hepatic retinoid stores and increased brown adipose retinoid levels, which were linked to significant decreases in body weight and fat mass in chow-fed UCP1-RBP4 mice compared with littermate controls. Compared with control mice, UCP1-RBP4 mice presented moderate increases in whole-body energy expenditure and thermogenesis when subjected to cold exposure. This improvement correlated with increased levels of lipolysis and FAO in BAT, facilitated by the activation of canonical adrenergic signaling. Moreover, the present findings support the notion that substantial RBP4 expression and release occur in BAT, especially after thermogenic activation. Notably, our observations revealed that BAT activation due to the overexpression of RBP4 influences white fat browning. This was evidenced by the presence of UCP1-positive beige fat and the expression of other genes associated with BAT in the SubQ of UCP1-RBP4 mice.

Previous studies by Kiefer and colleagues revealed that systemic levels of vitamin A and its transporter, RBP4, are increased during cold exposure. They demonstrated that cold exposure leads to a shift in retinol abundance from the liver toward the SubQ rather than the BAT, which correlates with increased thermogenic gene transcription in the SubQ. Additionally, using *Rbp4*^−/−^ mice provided evidence that RBP4 is necessary for cold-mediated adipose tissue browning and thermogenesis^24^. We have also reported that brown adipose RBP4 mediates shifts in retinol levels among the liver, BAT and plasma, which are accompanied by elevated circulating levels of RBP4. This hepatic redistribution of retinol toward BAT suggests that the increase in circulating retinol levels may lead to increased retinol uptake and accumulation in RBP4-overexpressing BAT, where it could be stored as retinyl esters within the fat depot of BAT. However, to substantiate this hypothesis in vivo, retinoid-tracing studies using [^3^H]-retinol are needed. Consequently, BAT may serve as an augmented source of retinol and RBP4 proteins in response to thermogenic stress. Villarroya and colleagues previously proposed that enhanced lipolysis following a thermogenic stimulus could lead to increased hydrolysis of retinyl esters within the fat depots of BAT. This process could result in the release of retinol, potentially necessitating the coordinated synthesis of the RBP4 protein to bind it^36^. Our findings somewhat agree with this scenario. Accordingly, this may explain why Kiefer and colleagues did not observe an abrogation of cold-mediated changes in *Rbp4*-deficient BAT, unlike that observed in WAT^24^.

In addition to classic BAT, beige adipocytes that emerge within WAT upon cold stimulation also have a high thermogenic capacity. As RBP4 is expressed in both brown and beige adipocytes, we investigated whether it plays a similar role in beige adipocytes as it does in brown adipocytes via ex vivo beige adipocytes of isoproterenol-stimulated SubQ from the control and UCP1-RBP4 mice. Upon isoproterenol stimulation, beige adipose tissues from the UCP1-RBP4 mice presented PKA activation (data not shown) and subsequently increased lipolysis rates compared with those from the control mice, suggesting that it acts in a similar manner in brown adipocytes. Housing mice at 20–22 °C causes moderate thermogenic stress. Immunohistochemistry images revealed that some UCP1-positive cells, as well as the lipid droplet size and BAT-like morphology, occurred in the SubQ of the chow-fed UCP1-RBP4 mice compared with the control SubQ. These results suggest that the overexpression of BAT-specific RBP4 activates the thermogenic program in WAT and affects systemic metabolism. Increased retinoic acid signaling contributes to thermogenic functions, as is widely reported^28,37,38^, and retinoic acid target genes are markedly induced in retinol-stimulated human adipocytes^24^. Given that blunted WAT browning and the thermogenic capacity in the *Rbp4*-deficient mice were attributed to alterations in retinoid shuttling toward adipose tissue^24^, we cannot rule out the possibility that retinol-bound RBP4 released from the UCP1-RBP4 mice facilitates WAT browning. However, further research is needed to establish whether retinol is secreted from brown fat bound to RBP4 and how brown fat-released RBP4 affects this process. In this context, observing the phenotype of UCP1-RBP4 mice when they consume a vitamin A-deficient diet would be intriguing. Alternatively, it is possible that an unknown factor produced by RBP4-driven BAT activation could have induced these changes. Wang et al. reported that retinoic acid promotes WAT browning by activating the binding of vascular endothelial growth factor (VEGF) signaling to retinoic acid receptor and RXR nuclear receptors. In addition, retinoic acid activates the VEGF–VGFR2 pathway, stimulating the proliferation of platelet-derived growth factor receptor α-positive adipose precursor cells. It has been suggested that increased angiogenesis enhances the energy expenditure of active adipose tissue, resulting in WAT browning^39^.

In BAT, β3-AR-induced lipolysis supplies fuel for oxidative metabolism, and cold exposure accelerates the clearance of TGs from the bloodstream by increasing the activity of lipoprotein lipase and facilitating their uptake into brown adipocytes^40^. The overexpression of brown adipose RBP4 increased systemic lipolysis in basal-state mice; however, this did not increase plasma FFA levels. However, when ex vivo brown fat explants from 3-month-old male UCP1-RBP4 mice were examined, significant FFA release was observed. Interestingly, an inconsistency was noted, as fasting plasma FFA levels were markedly decreased in the 6–7-month-old male UCP1-RBP4 mice compared with the control mice. There are several possible explanations for this: (1) FFA release from brown adipocytes could be well balanced with its uptake by peripheral tissues; (2) FFAs could be reutilized by BAT through a futile re-esterification cycle; or (3) FAO could be increased in the BAT of the UCP1-RBP4 mice. Of these, the former two seem unlikely, given the lower hepatic and comparable brown fat TG levels observed between the genotypes. The increased oxygen consumption and heat production in the aged UCP1-RBP4 mice suggest the oxidation of these lipids, which explains the reduced adiposity and whole-body energy homeostasis observed in the UCP1-RBP4 mice. However, we did not observe any increase in UCP1 expression in the BAT of the UCP1-RBP4 mice, although the levels of the phosphorylated forms of CREB, a well-known transcription factor for UCP1 expression, were increased in the UCP1-RBP4 mice. It is likely that RBP4-mediated brown fat activation did not occur sufficiently to increase UCP1 expression; FFAs could be released through lipolysis and may solely serve as substrates for β-oxidation. Previous studies have shown that increased *Ucp1* mRNA expression is induced by a large dose of retinoic acid (intraperitoneal injection of 100 mg/kg body weight); thus, increased retinol levels in the BAT of the UCP1-RBP4 mice might have been insufficient to induce UCP1-mediated thermogenesis^41^.

Changes in lipolytic activity can influence the regulation of genes associated with brown fat activation and mitochondrial function^7^. Our findings demonstrate that increased lipolysis in UCP1-RBP4 mice relies on the upregulation of *Adrb3* mRNA, cAMP production, PKA activation and phosphorylation of HSL and CREB in these mice, as well as on RBP4 loss of function in brown adipocytes. Thus, RBP4 positively activates the canonical adrenergic signaling pathway in brown adipocytes in a cell-autonomous manner. Although lipolysis and mitochondrial function are regulated by RBP4, the magnitude of the difference is not significant at the basal level. However, when cold stimulation was introduced, the discrepancy increased and UCP1-mediated thermogenesis was induced, suggesting an additional mechanism beyond RBP4-mediated regulation. Thyroid hormone signaling is also critical for regulating energy expenditure and BAT activation^42^. Studies have shown that elevated cAMP levels induce DIO2 expression, converting T4 to T3. The subsequent increase in intracellular T3 levels positively stimulates cAMP production, establishing a feedback loop in which cAMP, DIO2 and T3 mutually reinforce each other. In addition, DIO2 is associated with *Ucp1* gene expression because the thyroid hormone-response elements are located in the 5′-flanking region of *Ucp1* (ref. ^43^). Accordingly, RBP4-mediated *Dio2* upregulation or downregulation suggests a positive correlation between RBP4 and thyroid hormones, and the synergy of both stimuli might provide an adequate BAT effect.

Even subtle alterations in the tissue retinoid content can exert significant functional effects, particularly at the transcriptional level^44^. Consequently, the increased total retinol levels observed in the RBP4-overexpressing BAT probably affect the transcriptional regulation of genes linked to brown fat activation and adaptive thermogenesis. Network analysis based on RNA sequencing highlighted the substantial upregulation of genes associated with fatty acid elongation and lipid metabolism pathways. In BAT, adequate lipid turnover is pivotal, and maximal thermogenesis relies on maintaining a pool of long-chain fatty acids. Published studies have indicated that upon cold challenge, the expression levels of genes involved in long-chain fatty acid procurement and combustion increase^45^. Notably, in the *Elovl3*-ablated mice, the inability to hyperactivate BAT in response to cold exposure led to a reliance on muscle shivering to maintain body temperature^46^. Therefore, the upregulation of *Elovl3* in the UCP1-RBP4 mice suggests that increased lipolysis, as previously demonstrated, prompts an increased demand for fatty acids, thus stimulating the expression of *Elovl3* to replenish the intracellular levels of very long-chain fatty acids. Furthermore, the upregulation of *Acot1* contributes to increased β-oxidation as it enzymatically breaks down long-chain acyl-CoA into FFAs, providing mitochondria with the necessary substrates for FAO^47^. Intriguingly, our network analysis revealed increased levels of the mitochondrial gene *Bnip3*. A published study revealed the existence of a novel PPARγ–Bnip3 axis that regulates mitochondrial network fragmentation in adipocytes, leading to increased adipose mitochondrial bioenergetics, fatty acid utilization and insulin-stimulated glucose disposal, which is attributed to its profission activity^48^. Additionally, DEPP1-triggered reactive oxygen species (ROS) accumulation induces FGF21 expression, which mediates increased FAO and ketogenesis^49^. Another noteworthy observation in RBP4-overexpressing brown adipocytes is the upregulation of *Nr1h3*. NR1H3 (liver X receptor α; LXRα) is strongly activated by 9-*cis* retinoic acid and is essential for transactivation through a distinct retinoid response element that interacts with endogenous RXR^50^. LXRα has been reported to be a direct transcriptional inhibitor of the β-AR-mediated cAMP-dependent *Ucp1* gene by binding to the critical enhancer region of the *Ucp1* promoter^51^. These findings suggest that LXRα may counterbalance negative regulatory mechanisms in RBP4-mediated thermogenesis. Interestingly, Yoo et al.^52^ reported that 9-*cis* retinoic acid is present in BAT at levels comparable to ATRA. Fasting increased the concentration of 9-*cis* retinoic acid in BAT by approximately 1.7-fold, whereas ATRA levels remained unchanged^52^. These findings emphasize the importance of 9-*cis* retinoic acid in the metabolic regulation of BAT. The production of 9-*cis* retinoic acid is also linked to MOCS1 and MOCS3, genes critical for the synthesis of the molybdenum cofactor, which is essential for the activity of aldehyde oxidase (AOX1)^53,54^. AOX1, in turn, catalyzes the conversion of 9-*cis* retinal to 9-*cis* retinoic acid, linking these genes to retinoid metabolism. Although direct experimental evidence for the role of AOX1 in converting 9-*cis* retinal is lacking, its confirmed involvement in converting all-*trans* retinal to ATRA supports this possible pathway^55^. LXRα activation also induces *Abcg1*-mediated cholesterol efflux^56^, *Cidec*-regulated TG accumulation^57^ and increased LPL activity^58^. Overall, the overexpression of RBP4 in BAT is intricately involved in a complex gene network that directly and indirectly influences thermogenesis. However, further research is imperative to identify the specific target genes strongly regulated by RBP4.

In summary, the overexpression of brown adipose RBP4 positively regulates canonical adrenergic signaling, affecting brown fat activation, including lipolysis, and enhances WAT browning. This leads to increased energy expenditure and improved thermoregulation. Additionally, this is associated with altered systemic retinoid homeostasis between the liver and BAT through changes in circulating retinol and RBP4 levels. Thus, our findings establish that RBP4 has distinct tissue-specific roles that affect whole-body energy metabolism. Brown adipose RBP4 regulates systemic lipid and glucose homeostasis and, if targeted in a brown adipose-specific manner, it may present a therapeutic approach for metabolic abnormalities.

## Supplementary information

Supplementary Information
