# Supplementary material for: The brown fat-specific overexpression of RBP4 improves thermoregulation and systemic metabolism by activating the canonical adrenergic signaling pathway
Source: Exp Mol Med. 2025 Mar 3;57(3):554–66. doi: 10.1038/s12276-025-01411-6 (PMC11958748; doi:10.1038/s12276-025-01411-6)
Supplement: Supplementary file 1 — Supplementary Information [file 12276_2025_1411_MOESM1_ESM.pdf]

Supplementary Information

The brown fat-specific overexpression of RBP4 4 improves thermoregulation and systemic metabolism by activating the canonical adrenergic signaling pathway

Jong Yoen Park[1*], Eun Sun Ha[1*], Jimin Lee[2], Pierre-Jacques Brun[3], Yeri Kim[1], Sung Soo Chung[4], Daehee Hwang[2,5], Seung-Ah Lee[6†], Kyong Soo Park[1,6†]

Experimental Procedures

Metabolic measurements

Metabolic parameters were determined using the Oxymax Comprehensive Laboratory Animal Monitoring System (Oxymax-CLAMS; Columbus Instruments, Columbus, OH, USA), which is an automated open-circuit indirect calorimetry system that was placed in an environmentally controlled cabinet. The calorimetry system simultaneously measured O_2 consumption, CO_2 production, respiratory exchange rate, heat or energy expenditure, locomotor activity, and food consumption. Body composition was measured in non-anesthetized mice using EcoMRI (Bruker Minispec LF50; Bruker, Billerica, MA, USA). For glucose tolerance test, mice were fasted overnight and subsequently administered intraperitoneal injections of a glucose challenge (2 g glucose/kg body weight). Blood glucose levels were measured using a standard glucometer (Accu-Chek Active; Roche Diagnostics, Mannheim, Germany) at the indicated time points up to 2 h after injection. A cold challenge was performed at 4°C for up to 6 h, and the rectal temperature of mice assessed at the indicated time points using a rectal probe.

Cold-challenge

Male C57BL/6J mice, aged 23 weeks, were used in this study. Cold exposure was conducted in temperature-controlled chamber, with continuous monitoring to ensure stable environmental conditions. Following a 6-h fasting period, the mice were individually housed in separate cages for the cold exposure experiment set at 4 ºC. All enrichment materials, except bedding, were removed, and food was restricted while water remained accessible throughout the experiment. Rectal temperature was measured at 0, 1, 2, 4, and 6 h during the 6-h cold challenge using a digital thermometer. To minimize handling stress, each temperature measurement was completed within 15-30 seconds per mouse.

Biochemical analyses

Commercial kits were used to assess plasma, hepatic, and brown adipose triglyceride (TG) levels (Cayman Chemical, Ann Arbor, MI, USA), along with plasma FFA (Biovision, Mipitas, CA, USA), leptin (Abcam, Cambridge, UK), and adiponectin (Abcam) levels, according to the manufacturers' instructions.

Immunohistochemistry

Tissue sections were fixed overnight in 10% (v/v) neutral-buffered formalin, embedded in paraffin, sectioned, and stained with hematoxylin and eosin. All histological examinations were performed at the Pathology Core Facility at the Seoul National University Hospital Biomedical Research Institute (Seoul, Republic of Korea), and images captured using an ECLIPSE Ci-L microscope (Nikon Instruments Inc., Melville, NY, USA).

Cell culture

Murine brown adipocytes (kindly gifted by Dr. Shingo Kajimura, Harvard Medical School, Boston, MA, USA) were cultured in Dulbecco's Modified Eagle Medium with high glucose (Hyclone Laboratories Inc., Logan, UT, USA) supplemented with 10% fetal bovine serum (Thermo Fisher Scientific, Waltham, MA, USA) and 1% penicillin-streptomycin (Thermo Fisher Scientific) in a humidified atmosphere of 5% CO_2 at 37°C. Two days after reaching confluence, the cells were induced to differentiate using a specific medium comprising 5 µg/mL insulin (Sigma-Aldrich), 1 nM 3,3′,5-triiodo-L-thyrosnine (T3) (Sigma-Aldrich), 0.125 mM indomethacin (Sigma-Aldrich), 2 µg/mL dexamethasone (Sigma-Aldrich), and 0.5 mM IBMX (Sigma-Aldrich) (day 0). After two days, the medium was replaced with Dulbecco's Modified

Eagle Medium supplemented with 5 μg/mL insulin in 10% fetal bovine serum and antibiotics (day 2), and replaced every two days from days 4–8. For knockdown experiments, 100 nM siRNA (Dharmacon ON-TARGET plus Mouse RBP4 #19662; Dharmacon, Lafayette, CO, USA) was reverse-transfected using Lipofectamine RNAiMAX (Invitrogen, Waltham, MA, USA) on day 4 of differentiation and harvested on day 6. Primary brown pre-adipocytes were prepared from 7-week-old male UCP1-RBP4 mice and their matched control mice. The interscapular BAT was isolated, minced, and then incubated in digestion buffer (phosphate-buffered saline with 10 mM $CaCl_2$) containing 1.5 U/mL collagenase D (Roche, Basel, Switzerland) and 2.4 U/mL dispase II (Roche) in a 37°C shaking incubator for 30 min. Mature adipocytes and connective tissues were separated from the cell pellet via centrifugation at 800 × *g* for 10 min at 4°C. The cell pellet was then suspended in RBC lysis buffer (Sigma-Aldrich) and filtered through a 70-μm mesh filter (BD Biosciences, Franklin Lakes, NJ, USA). The pelleted stromal vascular cells were resuspended in Dulbecco's Modified Eagle Medium containing 10% fetal bovine serum and antibiotics and seeded in 100 mm dishes for adipogenic differentiation, as mentioned previously.

Western blotting

Lysates of tissues or brown adipocytes were prepared in radioimmunoprecipitation assay buffer (Thermo Fisher Scientific) containing a protease inhibitor cocktail (Thermo Fisher Scientific). Protein samples (25~40 μg) were subjected to sodium dodecyl sulfate-polyacrylamide gel electrophoresis, transferred onto a nitrocellulose membrane (GE Healthcare, Chicago, IL, USA), and immunoblotted using the primary antibodies listed in Supplementary Table. For plasma RBP4 and transferrin levels, each lane was loaded with 0.1 μL of plasma. Protein bands were visualized using an enhanced chemiluminescence kit (Thermo Fisher Scientific), and the

polyclonal anti-mouse γ-tubulin antibody (1:5000; Sigma-Aldrich #T6557) was used as a loading control.

Quantitative real-time PCR

Total RNA was isolated from tissues or cells using TRIzol (Life Technologies, Waltham, MA, USA) and reverse-transcribed to cDNA using SuperScript II Reverse Transcriptase (Promega, Madison, WI, USA), according to the manufacturer's instructions. Quantitative real-time PCR (qPCR) was performed using SYBR Premix Ex Taq (Takara, Shiga, Japan) with an ABI 7500 Real-Time PCR System (Applied Biosystems, Foster City, CA, USA). Each C*t* value was subtracted from the 36*B*4 C*t* value of the same samples (dC*t*) and then from the dC*t* value of each control set (ddC*t*). Relative mRNA expression levels are expressed as $2^{-\Delta\Delta Ct}$. The primer sequences used are available upon request.

Seahorse assay

The oxygen consumption rate (OCR) was measured using a Seahorse XF96 Extracellular Flux Analyzer (Agilent Tech, Santa Clara, CA, USA), according to the manufacturer's protocol. Briefly, cells were seeded two days before the assay at a density of 2×10^4 cells/well in a 0.2% gelatin-coated XF96 microplate. One day before the assay, 0.1 μM isoproterenol was added to the XF96-well plate, and 200 μl calibration buffer added to the XFe96 assay cartridge, which was then equilibrated in a 37°C non-CO_2 incubator. For OCR measurements, the culture medium was replaced with 180 μL/well Agilent Seahorse XF Base Medium (pH 7.4) with 10 mM glucose, 1 mM sodium pyruvate, and 2 mM glutamate, and the chemical compounds added to the assay cartridge drug port. Measurements were taken at 6 min intervals with three measurements, after injection of the compounds affecting bioenergetics: 1 μM oligomycin

(Sigma-Aldrich), 1 μM carbonyl cyanide-*p*-(trifluoromethoxy)phenylhydrazone (FCCP) (Sigma-Aldrich), 2.5 μM rotenone (Sigma-Aldrich), and 1.25 μM antimycin A (Sigma-Aldrich). Cell count was performed post-measurement, and the OCR value per 1,000 cells thereafter calculated.

Statistical analysis

All data are given as the means ± SEM, if not specified otherwise. Comparisons between two groups were performed using an unpaired Student's *t*-test. A P-value < 0.05 was considered statistically significant.

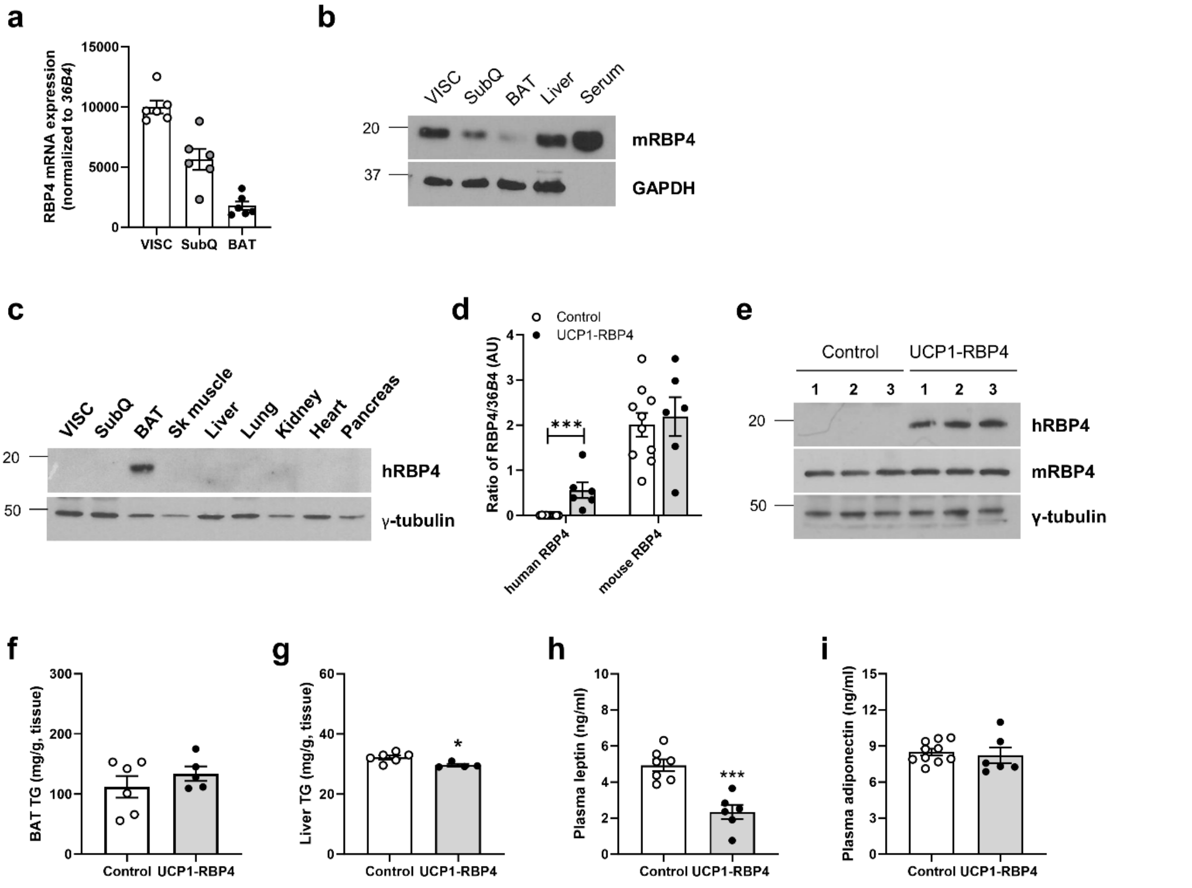

Supplementary Fig. 1. Characterization of UCP1-RBP4 mice fed a chow diet. (a) qPCR analysis of the relative mRNA expression of endogenous mouse RBP4 (*Rbp4*) in VISC, SubQ, and BAT of C57BL/6J mice (*n* = 5). (b) Immunoblots showing relative mRBP4 protein expression in VISC, SubQ, BAT, liver, and serum. (c) Immunoblots showing human RBP4 (hRBP4) protein expression in tissue homogenates prepared from UCP1-RBP4 mice. (d) qPCR analysis of the relative mRNA expression of the human and mouse RBP4 gene in the BAT of UCP1-RBP4 and matched control mice (*n* = 6–10). (e) Immunoblots showing hRBP4 and mRBP4 protein expression in the BAT of three UCP1-RBP4 and three littermate control mice. BAT triglyceride (TG) (f), liver TG (g), plasma leptin (h), and plasma adiponectin (i) levels of 6- to 7-month-old male UCP1-RBP4 and matched control mice following overnight fasting (*n* = 5–10). Data are presented as mean ± SEM. **P* < 0.05; ****P* < 0.001.

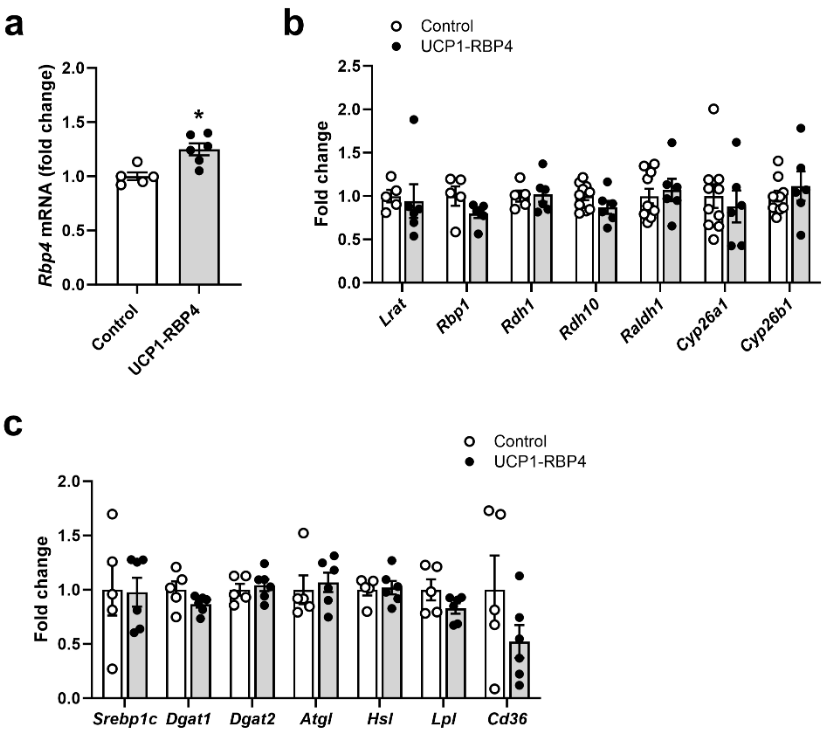

Supplementary Fig. 2. Brown adipose RBP4 overexpression induces *Rbp4* mRNA expression, but not gene associated with retinoid machinery and lipid metabolism in the liver of mice. (a) qPCR analysis of the mRNA expression of *Rbp4* in the liver in UCP1-RBP4 and matched control mice fed a chow diet. qPCR analysis of the relative mRNA expression of genes related to retinoid machinery (b) and lipid metabolism (c) in the liver (n = 5–10). Data are presented as mean ± SEM. *$P < 0.05$.

Supplementary Table. List of the antibodies used in this study.

Primary antibodies	Company (Catalog #)	Dilution
phospho-PKA substrate	Cell Signaling Technology (#CST 9624S)	1:3,000
phospho-HSL (Ser660)	Cell Signaling Technology (#CST 45804)	1:500
total-HSL	Thermo Fischer Scientific (#PA5-17186)	1:1,000
phospho-CREB (Ser133)	Cell Signaling Technology (#CST 9198S)	1:1,000
total-CREB	Abcam (#ab32515)	1:1,000
human RBP4	SinoBiological, Inc. (#10354-RP02)	1:1,000
mouse RBP4	SinoBiological, Inc. (#50170-RP02)	1:1,000
transferrin	Bethyl lab (#A90-129A)	1:10,000
